# Effect of a School-Based Hygiene Behavior Change Campaign on Handwashing with Soap in Bihar, India: Cluster-Randomized Trial

**DOI:** 10.4269/ajtmh.18-0187

**Published:** 2018-08-13

**Authors:** Henrietta E. Lewis, Katie Greenland, Val Curtis, Wolf-Peter Schmidt

**Affiliations:** London School of Hygiene and Tropical Medicine, London, United Kingdom

## Abstract

Changing hand hygiene behavior at scale in the community remains a challenge. The objective of this study was to estimate the effect of Unilever’s school-based “School of 5” handwashing campaign on handwashing with soap (HWWS) in schoolchildren and their mothers in the Indian state of Bihar. We conducted a cluster-randomized trial in two districts. We randomized a total of 32 villages with at least one eligible school to intervention and control groups (1:1) and recruited 338 households in each group for outcome measurement. We used structured observation in households to measure HWWS at target occasions (after defecation, soap use during bathing, and before each main meal) in schoolchildren and their mothers. Observers were blinded to intervention status. We observed 636 target occasions (297 in the intervention arm and 339 in the control arm) in mothers and school-going children. After the intervention, HWWS prevalence at target occasions was 22.4% in the control arm and 26.6% in the intervention arm (prevalence difference +4.4%, 95% confidence interval: −4.0, 12.8). The difference was similar in children and mothers. Observers appeared to be adequately blinded to intervention status, whereas observed households were successfully kept unaware of the purpose of observations. To conclude, we found no evidence for a health-relevant effect of the School of 5 intervention on HWWS in schoolchildren and their mothers. Qualitative research suggested that reasons for the low impact of the intervention included low campaign intensity, ineffective delivery, and a model possibly not well tailored to these challenging physical and social environments.

## INTRODUCTION

Handwashing with soap (HWWS) may substantially reduce morbidity and mortality from infection spread by fecal–oral routes and person-to-person contact, including gastrointestinal infections,^1,2^ respiratory infections,^3,4^ trachoma,^5^ fatal neonatal infections,^6^ and worm infections.^7^

Although knowledge on the health benefits of handwashing appears widespread, prevalence of adequate handwashing is low.^8,9^ Interventions to increase HWWS have produced varying results,^10–13^ but many fail to generate relevant behavior change or health benefits when applied at scale.^14^

As well as offering a useful delivery platform facilitating scalability, basing behavior change interventions within the educational setting of schools has been suggested as an important channel to change behavior, as many children’s habits and behaviors may be learned at school. Programs that use schools for the delivery include interventions promoting physical activity and healthy eating, and condom use.^15,16^ School-based water, sanitation, and hygiene interventions have been associated with improvements in health.^7,13,17^ Programmatically, citing children as “agents of change”^18,19^ has become popular, but while there is some suggestion that healthy practices may be transferred to family members in the home,^20,21^ rigorous studies have not yet been conducted to prove this concept.

Using a combination of emotional drivers and conventional educational methods, the Unilever Lifebuoy School-Based Handwashing campaign (“School of 5”) aims at increasing HWWS among schoolchildren and their mothers. An intensive 40-week version of the program with more than 20 visits to school and households was tested in a cluster-randomized trial (CRT) in Mumbai, India. The intervention was associated with a 25% reduction in reported diarrhea in children less than 5 years of age living in families with children attending intervention schools.^21^ In collaboration with the U.K.-based charity Children’s Investment Fund Foundation (CIFF), Unilever rolled out a shorter version of the campaign, delivered over 21 days during just four school visits and no home visits, across the Indian state of Bihar. The specified aim of the program was to reduce diarrhea morbidity and mortality in children less than 5 years of age.

This article reports the results of a trial to assess the effect of the short version of Unilever/Lifebuoy’s School of 5 campaign on HWWS in schoolchildren and their mothers at home at specified target occasions promoted by the intervention.

## METHODS

### Study design and randomization.

The study was conducted between April 2016 and January 2017 in two districts of Bihar (Samastipur and Vaishali). The study districts were chosen based on reasonable proximity of the districts to Patna, the state capital, while still representing a typical rural setting in the state. The research was conducted as a CRT. Randomization was carried out at the village level. All eligible schools in a village either received the intervention or were allocated to control (no intervention). Schools were eligible to receive the intervention if they were government schools and had at least 150 children enrolled.

The research was designed to occur over three phases: 1) randomization, 2) estimation of behavior change, and 3) estimation of health effect. In the first phase, we selected eight blocks (an administrative unit below district) within the two districts where the implementer had not yet delivered the intervention. All villages with eligible schools in these eight blocks were randomized to intervention (169 villages) or control (*N* = 170 villages). Randomization was carried out stratified by the number of eligible schools per village, as a proxy of village size. The four size strata were as follows: 1) villages with one school (*N* = 149), 2) two schools (*N* = 70), 3) three or four schools (*N* = 60), and 4) five or more schools (*N* = 60). Randomization was further stratified by block to ensure similar numbers of intervention and control schools in each of them. Randomization was carried out by the trial statistician (W-P. S.) using a random number generator in Excel.

**Figure 1.**
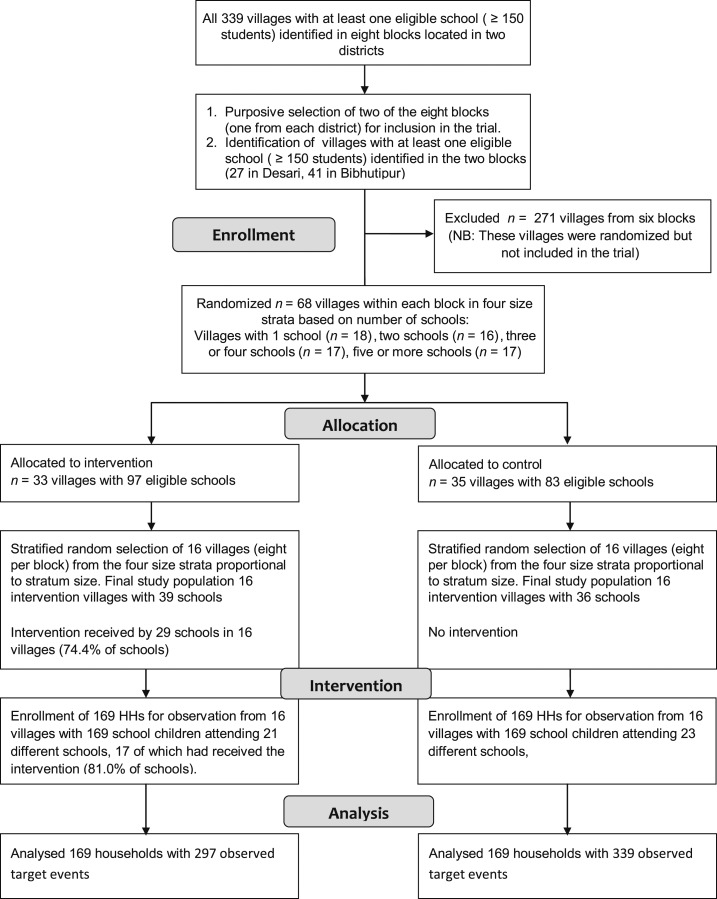
CONSORT flow diagram.

For the estimation of behavior change reported here, we purposely selected one block from each district (based on logistics) to measure the effect of the intervention on HWWS. Within each of these two blocks (Bibhutipur and Desari), 16 villages were selected at random proportional to stratum size within strata, from the total of 41 villages in Bibhutipur and 27 villages in Desari (Supplement Table 1). Figure 1 shows the CONSORT flow diagram for the trial. No baseline observation data were collected to decrease the risk of reactivity and responder bias. The trial manager and the study team were blind to village allocation status.

To limit their ability to concentrate campaign efforts in a small number of villages, the implementers were kept unaware of the blocks and villages in which the effect on HWWS would be assessed. The implementers were aware that in the third step, the health effect of the intervention might eventually be measured in all eight blocks. However, because we found little evidence for behavior change in the second step, the campaign was terminated across Bihar. The research did not proceed to the third phase, and no health outcomes were measured. This article only concerns the second phase, that is, the effect of the intervention on behavior.

### The intervention.

Unilever’s “School of 5” program, based on the company’s antibacterial soap Lifebuoy, aimed to reach at least nine million schoolchildren across Bihar state. The program aimed to change social norms in the target children and create peer pressure, using the fear of contamination and disgust to encourage adoption of HWWS as a routine behavior in schools and at home.

The campaign focused on avoiding germs by HWWS, preferably Lifebuoy, at five specific occasions: before each meal (breakfast, lunch, and dinner), after defecation, and while bathing. “School of 5” was designed to appeal to the campaign’s direct target audience, 6–12 years old children, and their mothers or other primary female caretakers.

An overview of the core 21-day behavior change program as delivered across Bihar is shown in Table 1. Teams of two promoters and one supervisor used by a “brand activation agency” visited each school four times over the course of 21 days (one visit per week), with teachers and pupils encouraged to conduct ongoing activities between team visits. Each block was served by a different team of promoters. Depending on school size, promoters ran three to four sessions per visit, in batches of around 100 children per session, to reach all of the target children in the school. The team of promoters engaged the students in activities to create awareness and encourage commitment, coupled with activities meant to reinforce core messages and reward active participants. The children were also given 10 “enrollment cards” for telling up to 10 relatives or friends what they learned during the sessions and having them pledge to also HWWS at the target occasions. During the first visit, children were given invitations for their mothers to attend a “mothers’ meeting” held once during the 21-day period, at an *aganwadi* (childcare) center or the school.

**Table 1:** Intervention content

Activities	Content	Purpose
Week 1		
Meet with principal	Meet with principal and/or headmaster to introduce So5 program	Establish the first point of contact with school
	Show authorization letter from the state government	Demonstrate government support of program
	Principal or headmaster is photographed granting permission to conduct the program within their school	Obtain permission to conduct sessions; legitimize program; gain support of influential leaders
Put-up posters	Hang three posters on school grounds, including near hand pump, that describe handwashing steps and critical times for HWWS	Legitimize campaign; generate interest about content; establish HWWS as a social norm
Conduct first school visit	Introduce So5 program	–
	Describe five target occasions and introduce superheroes	Create awareness about critical HWWS times; generate interest for future sessions
	Teach handwashing steps and actions	Use memorable phrases and actions to make the behavior more engaging; reinforce key program messaging
	Teach and have children take handwashing pledge	Secure commitment to adopting and regularly practicing HWWS
	Distribute and explain daily diary task	Serve as reminder to practice HWWS daily, during five target occasions
	Appoint class monitors to supervise HWWS during midday meal	Encourage accountability and adherence to HWWS during midday meal
	Distribute Lifebuoy soap to teachers during midday meal and supervise HWWS	Enable soap use during midday meal; live action at hand pump to aid remembering handwashing steps
Conduct mothers’ mapping	Go door to door in villages near to program school to invite mothers or other female caregivers to mothers’ meeting the next week	Raise awareness of So5 and increase attendance for the mothers meeting; generate interest about program messaging
Week 2		
Conduct second school visit	Recap five target occasions, superheroes, handwashing steps, and pledge	Reiterate campaign’s key messaging and its vehicles
	Check daily diaries for the past week	Serve as a reminder to practice HWWS daily, during five target occasions
	Flip chart presentation of first and second	Present stories about superheroes and their handwashing-related adventures; highlight the importance of HWWS through narratives
	Reward one to two students who can recall steps and superheroes	Encourage other students to pay attention to and participate in sessions
	Conduct glitter ball demonstration	Create awareness about germ theory in a fun manner
Conduct mothers’ meeting	Introduction to So5	Remind female caregivers to HWWS before preparing food, feeding children, and during five target occasions
	Flip chart story presentation (mom-specific story)	Show potential for child health and accomplishments, as linked to HWWS behavior
	Conduct glitter ball demonstration (either with two to four moms or children)	Create awareness about germ theory in a fun manner
	Teach and have mothers take handwashing pledge	Secure commitment by female caregivers to adopting the behavior, while establishing it as a social norm
Week 3		
Conduct third school visit	Recap five target occasions, superheroes, handwashing steps, and pledge	Reiterate campaign’s key messaging and its vehicles
	Check daily diaries for past week	Serve as a reminder to practice HWWS daily, during five target occasions
	Flip chart presentation of third and fourth stories about superheroes and their handwashing-related adventures	Present stories about superheroes and their handwashing-related adventures; highlight the importance of HWWS through narratives
Week 4		
Conduct fourth school visit	Recap five target occasions, superheroes, handwashing steps, and pledge	Reiterate campaign’s key messaging and its vehicles
	Check daily diaries for past week	Serve as a reminder to practice HWWS daily, during five target occasions
	Award three to four students (per school) with stickers and comic books at the end of the program	Provide positive reinforcement for students most active during sessions and in completing the diary

HWWS = handwashing with soap; So5 = School of 5.

### Recruitment of participants.

The outcomes were assessed at household level. The eligibility criteria for enrolled households were as follows: 1) presence of a child regularly attending an eligible school in the previous 3 months and 2) presence of a younger sibling less than the age of 5 years in the same household. To avoid unblinding of the study team, eligible households in each village were identified by house-to-house search, rather than by visiting the schools to obtain a list of pupils. Because the number of eligible households per village was low in most villages (usually less than 20), and to simplify procedures, enrollment was done until the intended number of houses was reached (*N* = 12), or no further eligible houses could be found.

### Structured observation.

We used young, female enumerators to carry out all data collection including observation, following methods used in previous studies in similar settings.^10,11^ The structured observations were conducted in each village in a single morning. Enrolment of eligible study households occurred 1–3 days before the actual day of observation. All observations occurred early in the morning (starting between 5:00 and 6:00 am). On arrival at an enrolled house, the enumerator would place herself in a strategic location within the compound that allowed viewing of events as they occurred in the kitchen, and at the hand pump and latrine, if applicable. The enumerators used coded sheets to record their observations and short descriptions of observed occasions over a period of 3 hours. Participants were told that this was a study on domestic water use. Handwashing was not mentioned as a study aim. The observations were carried out at 8–10 weeks post-intervention to reduce the ability of the study population to link the study with the intervention.

Blinding of observers was assessed after each observation day in a village, by asking observers to guess retrospectively whether they thought the village in which the observations were carried out on that day was in the control or in the intervention arm (“do not know” was not allowed as an option). As observations in one village were carried out all on the same day (one per observer), each observer was asked to guess once for each of the 32 villages, resulting in a total of 338 observer/village combinations, equaling the number of enrolled households.

### Exposure survey.

Using unprompted and prompted questions, surveys were used to capture exposure to and recall of the intervention in all participating households 4–6 weeks after the structured observation. Mothers and children were interviewed simultaneously by two enumerators in different parts of the compound to reduce the chance that they would influence each other’s responses. Mothers were asked what they thought the purpose of the structured observation carried out 4–6 weeks earlier was. Social, demographic, and economic data were collected at the same time.

### Sample size and statistical analysis.

The primary outcome was HWWS at target occasions in children attending an eligible school and the mother or other female caretaker of that child (who was also the caregiver of a child less than 5 years of age). Target occasions were as follows: after defecation, (soap use) during bathing, and before each of three main meals (the “five occasions”). The primary outcome was a composite: we combined HWWS at target occasions with soap use during bathing, which was one of the five target occasions of the campaign, but technically is a different kind of behavior to observe. For all other four occasions (after defecation and before each of three meals), the outcome is based on whether a person practices HWWS at the occasion. By contrast, as bathing already is a hygiene behavior, the outcome is determined solely based on whether soap is used. Any observed bathing where soap is used meets the primary outcome definition. Handwashing activities such as after sweeping the house, cleaning the stove, handling livestock, washing utensils, or handwashing for no apparent reason were recorded as “handwashing at other times” and were not part of the primary outcome.

The sample size aimed at detecting a 15% point increase in HWWS at target occasions (from 5% to 20%), with 80% power resulting in 88 target occasions per arm. Allowing for a design effect of six because of within-household and within-village clustering of handwashing (as observed in a previous study in Andhra Pradesh)^10^ resulted in 528 observations per study arm. Based on our data from Andhra Pradesh, we expected five target occasions to be observed in school-aged children and mothers per 3-hour observation session. Recruiting 10 households per village resulted in 50 observations per village, that is, 11 villages per arm were required to observe more than 528 occasions per arm. We increased this number to 16 villages per arm as it became clear during the course of the study that intervention rollout was incomplete and the number of observed occasions per household was lower than that expected.

We used binomial regression analysis to calculate prevalence differences (binomial distribution, identity link). Clustering at village level was accounted for by using generalized estimating equations and robust standard errors. The main prespecified analysis was intention-to-treat. We built four additional models as sensitivity analysis of the primary end point. These models were not prespecified. First, we adjusted the analysis for location of the water source (for which there was some imbalance, Table 4). Second, because of incomplete rollout of the intervention, we calculated the complier average causal effect (CACE), which has been suggested as a method to address incomplete intervention uptake and contamination, while avoiding biases of per-protocol analysis.^22^ We used random allocation of the intervention as an instrumental variable for the target school-age child going to a school that actually received the intervention. We applied a two-stage least squares estimator and robust standard errors (to account for clustering by village), stratified by block (Desari versus Bibhutipur). Third, we adjusted this instrumental variable regression model for water source location. Fourth, we performed a similar instrumental variable regression with random allocation as instrumental variable for campaign exposure (defined as the target school-age child being able to describe the intervention). As we found some evidence for contamination in the exposure survey, we conducted post hoc geographic analysis. We recorded the Global Positioning System (GPS) location of every study school to explore the association between campaign exposure in control children and distance of their school to the nearest intervention school. Distance categories were chosen pragmatically to be meaningful and contain sufficient numbers. Statistical analyses were carried out in Stata 12.0 (StataCorp, College Station, TX). Geographic analysis was carried out in QGIS 2.1 (Quantum GIS).

### Role of the funding source.

The “School of 5” campaign in Bihar was funded by Unilever plc and the CIFF, a U.K.-based charity. This evaluation was funded by CIFF. The funders of this campaign and the study had no role in the data collection, data analysis, data interpretation, writing of the report, or decision to submit for publication.

### Ethics.

Study approval was granted by the ethics committee of the London School of Hygiene and Tropical Medicine and Hindustan Unilever’s Independent Ethics Committee (Bengaluru). The trial is registered at Clinicaltrials.gov (NCT02424812).

## RESULTS

Recruitment and outcome assessment occurred between July 15, 2016 and October 22, 2016 (8–10 weeks after completion of the intervention in a village). We recruited just more than 10 eligible households per village (10.6, standard deviation [SD] 1.2, range 9–12, no difference across arms). The mean age of the target children was 9.5 years in the control arm (SD 1.7, range 6–13), and 9.9 years in the intervention arm (SD 1.9, range 7–15). Table 2 shows that socioeconomic indicators were similar between control and intervention households. In both arms, most of the households were from other backward and scheduled castes (following a classification of castes used in India for administrative purposes). Most of the mothers had no education. More than 60% of households had electricity. Most households had a private tube well as their primary drinking water source. There was some imbalance with intervention households more often having the main water source outside the compound (40.2% in control, 48.8% intervention). The vast majority of households across arms practiced open defecation. Intervention rollout was incomplete in the 16 intervention villages: In 16.6% of enrolled households in the intervention arm, the target school-aged child went to an eligible school that for logistical reasons did not receive the intervention.

**Table 2:** Socioeconomic characteristics of intervention and control

	Control (*N* = 169)	Intervention (*N* = 169)
Household size, mean (SD)	7.1 (2.3)	7.1 (2.8)
Children less than 5 years of age, mean (SD)	1.4 (0.6)	1.5 (0.8)
Caste, *n* (%)		
General	17 (10.1)	9 (5.3)
Other backward caste	69 (40.8)	84 (49.7)
Scheduled caste	72 (42.6)	69 (40.8)
Scheduled tribe	7 (4.1)	5 (3.6)
Muslim	4 (2.4)	1 (0.6)
Other	0 (0)	1 (0.6)
Father’s education, *n* (%)		
None	62 (36.7)	59 (34.9)
Some primary	18 (10.7)	21 (12.4)
Primary completed	22 (13.0)	16 (9.5)
Some secondary	13 (7.7)	16 (9.5)
Secondary completed	21 (12.4)	15 (8.9)
Higher	33 (19.5)	41 (24.3)
Unknown	0 (0.0)	1 (0.6)
Mother’s education, *n* (%)		
None	117 (69.2)	99 (58.6)
Some primary	9 (5.3)	17 (10.1)
Primary completed	11 (6.5)	16 (9.5)
Some secondary	8 (4.7)	11 (6.5)
Secondary completed	6 (3.6)	13 (7.7)
Higher	18 (10.7)	12 (7.1)
Unknown	0 (0.0)	1 (0.6)
Electricity, *n* (%)	111 (65.7)	104 (61.5)
Motorbike, *n* (%)	15 (8.9)	15 (8.9)
House type, *n* (%)		
Pukka (concrete)	58 (34.3)	61 (36.1)
Semi-pukka (half concrete)	48 (28.4)	47 (27.8)
Kuccha (mud)	63 (37.3)	61 (36.1)
Drinking water source, *n* (%)		
Private tube well	105 (62.1)	102 (60.4)
Public tube well	42 (24.9)	47 (27.8)
Public tap	15 (8.9)	18 (10.7)
Dug well	4 (2.4)	1 (1.2)
Other	3 (1.8)	0 (0.0)
Location of water source, *n* (%)		
Inside house	60 (35.5)	45 (26.8)
Inside compound	41 (24.3)	41 (24.4)
Outside compound	68 (40.2)	82 (48.8)
Sanitation, *n* (%)		
Pour flush latrine	27 (16.0)	27 (15.0)
Pit latrine with slab	1 (0.6)	2 (1.2)
Pit latrine without slab	2 (1.2)	3 (1.8)
None	139 (81.2)	137 (82.0)

SD = standard deviation.

### Intervention effect.

We observed 4,533 events associated with hand hygiene, of which 2,595 were target events as defined by the School of 5 program (after defecation, before each meal, and bathing, Table 3). We observed 636 target events (297 in the intervention arm and 339 in the control arm) in mothers and school-going children (Table 4). Compared with control, the intervention was associated with a 4.4% point increase in HWWS, with a confidence interval (CI) crossing zero among schoolchildren and mothers (primary end point analysis). The difference between intervention and control was similar for mothers and children (Table 4). There was no evidence for an intervention effect in other person groups.

**Table 3:** Events observed

Event	Control		Intervention		Total	
	*N*	%	*n*	%	*N*	%
Before food preparation	263	8.6	259	8.9	522	8.7
Before eating a meal*	762	25.0	729	25.0	1,491	25.0
Before feeding a child	11	0.4	9	0.3	20	0.3
Before serving food	101	3.3	72	2.5	173	2.9
After latrine/defecation*	252	8.3	228	7.8	480	8.0
After cleaning a child	85	2.8	88	3.0	173	2.9
Soap use during bath*	228	7.5	225	7.7	453	7.6
Handwash at other times	1,351	44.3	1,312	44.9	2,663	44.6
Total	3,053	100	2,922	100	5,975	100

* Target occasion.

**Table 4:** Intervention effect

	Control		Intervention		Difference†	*P* value	95% CI	
	N*	HWWS	N*	HWWS			Lower	Upper
Schoolchildren and mothers (primary outcome)	339	22.4%	297	26.6%	4.4%	0.305	−4.0%	12.8%
Schoolchildren	261	19.5%	240	24.2%	4.6%	0.223	−2.8%	12.0%
Mothers	78	32.1%	57	36.8%	4.6%	0.658	−15.7%	24.8%
Nontarget school-aged children‡	329	16.4%	348	18.7%	2.6%	0.479	−4.6%	9.7%
Preschool children	418	10.8%	411	9.7%	−1.0%	0.760	−7.1%	4.6%
Men	124	30.7%	97	37.1%	7.4%	0.341	−7.8%	22.7%
All groups	1,242	18.2%	1,180	19.5%	1.5%	0.619	−4.5%	7.5%
Event type								
Before eating	163	0.0%	135	2.2%	2.2%	–	–	–
After defecation	103	27.2%	99	32.3%	3.6%	0.686	−13.8%	21.0%
During bath	73	65.8%	63	69.8%	4.2%	0.550	−9.7%	18.2%
Soap use at “other handwash”*	790	3.9%	763	5.8%	1.8%	0.066	−0.0%	3.6%
District								
Vaishali (Desari block)	180	23.3%	139	33.1%	10.1%	0.186	−4.9%	25.1%
Samastipur (Bibhutipur block)	159	21.4%	158	20.9%	−0.5%	0.869	−6.5%	5.5%

CI = confidence interval; HWWS = handwashing with soap.

* Number of observed target events.

† Prevalence difference adjusted for village-level clustering (generalized estimating equations/robust standard errors).

‡ School-aged children or siblings of target children living in the same compound but who went to an ineligible school, that is, they had no chance of receiving the intervention in either arm.

Soap use during bathing increased by 4.2% and HWWS after defecation by about 3.6%. We found no evidence for an increase in the proportion of “other handwashing events” where soap was used (as opposed to using only water, Table 4). Because each block had a different intervention team, we explored differences in the intervention effect across blocks. The intervention effect in Desari was +10%, whereas no effect was observed in Bibhutipur (test for interaction *P* = 0.217).

In the sensitivity analysis, when adjusting the primary end point analysis for water supply location (which was not well balanced across arms), there was a +6.1% point difference in HWWS between intervention and control (95% CI: −1.8%, 14.0%, *P* = 0.134). Using random allocation as an instrumental variable for a school receiving the intervention resulted in a prevalence difference of +5.3% (95% CI: −4.4%, 15.2%). Combining adjusting for water source location and the use of random allocation as an instrumental variable for a school receiving the intervention resulted in a prevalence difference of +7.0% (95% CI: 3.2%, 17.2%). Using random allocation as an instrumental variable for the schoolchild being able to describe the intervention resulted in a prevalence difference of +4.7% (95% CI: −9.2%, 18.7%).

### Exposure survey.

The intervention had no effect on disease risk perception in children (Table 5). Children within the intervention arm more frequently mentioned handwashing before eating and after defecation as a way of maintaining hygiene compared with those from the control arm. Only 21% of intervention children could correctly recall the five critical HWWS occasions. More than half of the children from the intervention arm (67.5%) were able to unambiguously describe the Lifebuoy School of 5 campaign. About 16% of control children appeared to have had some campaign exposure by being able to describe the campaign, suggesting contamination across arms. The percentage of control children able to describe the campaign strongly depended on the distance of their own school to the nearest intervention school (Figure 1).

**Table 5:** Exposure survey children

	Control		Intervention		Difference*	*P* value	95% CI	
	*N*	%	*N*	%			Lower	Upper
Children (all)	162	100.0	157	100.0	–	–	–	–
Health risk perception (unprompted)								
Mentions diarrhea as a health problem in village	4	2.5	3	1.9	−0.6%	0.677	−3.5%	2.3%
Mentions cough as a health problem in village	53	32.7	52	33.1	0.5%	0.937	−11.0%	12.0%
Mentions worrying about diarrhea	2	1.2	3	1.9	0.7%	0.608	−1.9%	3.2%
Mentions worrying about cough	45	27.8	47	29.9	2.1%	0.693	−8.4%	12.7%
The importance of hygiene and handwashing (unprompted)								
Mentions handwashing to keep healthy	28	17.3	28	17.8	0.5%	0.924	−10.4%	11.5%
Mentions handwashing as a method to be hygienic	39	24.1	47	29.9	6.4%	0.345	−6.9%	19.8%
Mentions using soap for handwashing	84	51.9	95	60.5	9.2%	0.235	−6.0%	24.4%
Mentions handwashing before eating	81	50.0	106	67.5	17.5%	0.002	6.5%	28.6%
Mentions handwashing after defecation	91	56.2	106	67.5	11.7%	0.117	−2.9%	26.3%
Mentions disease prevention as a reason for handwashing	89	54.9	78	49.7	−5.0%	0.424	−17.4%	7.3%
Mentions diarrhea prevention as a reason for handwashing	2	1.2	3	1.9	0.7%	0.636	−2.3%	3.7%
Hygiene advice (unprompted)								
Mentions receiving health advice from school	15	9.3	28	17.8	8.6%	0.011	2.0%	15.3%
Mentions being advised to wash hands	18	11.1	43	27.4	16.2%	0.001	6.4%	26.0%
Mentions being advised to wash hands with soap	12	7.4	36	22.9	15.4%	0.001	6.3%	24.5%
Exposure to campaign (unprompted)								
Describes school handwashing campaign	36	22.6	102	66.2	42.1%	< 0.001	27.4%	56.7%
Describes Lifebuoy/school of 5 campaign	26	16.4	104	67.5	49.4%	< 0.001	34.9%	63.8%
Describes song	9	5.6	65	41.4	35.1%	< 0.001	23.6%	46.5%
Describes names of handwashing superhero	5	3.1	55	35.0	31.6%	< 0.001	21.9%	41.2%
Describes mentioning of five target occasions	5	3.1	33	21.0	18.2%	< 0.001	11.9%	24.6%
Describes mentioning of handwashing with soap	11	6.8	28	17.8	10.8%	0.007	2.9%	18.8%
Describes campaign promoters in black/red shirts	3	1.9	22	14.0	12.3%	< 0.001	7.1%	17.4%

CI = confidence interval.

* Prevalence difference adjusted for village-level clustering (generalized estimating equations/robust standard errors).

As with children, the intervention had no effect on disease risk perception in mothers (Table 6). There was some indication for intervention mothers to more often mention soap use and handwashing before eating as ways to be hygienic. Only a few mothers in either arm recalled receiving health advice from their children. About 29% of mothers in intervention households had heard about a handwashing campaign having happened in the previous 3 months, whereas 17% specifically mentioned, without prompting, the Lifebuoy School of 5 campaign by name. Only a few mothers had heard of the mother’s meeting or had attended it.

**Table 6:** Exposure survey mothers

	Control		Intervention		Difference*	*P* value	95% CI	
	*N*	%	*N*	%			Lower	Upper
Mothers (all)	162	100.0	157	100.0	–	–	–	–
Health risk perception (unprompted)								
Mentions diarrhea as a health problem in village	13	8.0	11	7.0	−1.1%	0.748	−7.6%	5.5%
Mentions cough as a health problem in village	100	61.7	99	63.1	1.6%	0.834	−13.5%	16.7%
Mentions worrying about diarrhea in her children	12	7.4	11	7.0	−0.5%	0.874	−6.8%	5.8%
Mentions worrying about cough in her children	91	56.2	93	59.2	3.1%	0.595	−8.2%	14.3%
The importance of hygiene and handwashing (unprompted)								
Mentions handwashing to keep children healthy	26	16.1	16	10.2	−5.3%	0.24	−14.3%	3.6%
Mentions handwashing as a method to be hygienic	28	17.3	32	20.4	3.6%	0.525	−7.6%	14.8%
Mentions using soap for handwashing	85	52.5	94	59.9	8.8%	0.303	−7.9%	25.5%
Mentions handwashing before eating	67	46.9	87	55.4	8.8%	0.186	−4.3%	21.9%
Mentions handwashing after defecation	108	66.7	112	71.3	5.8%	0.464	−9.7%	21.2%
Mentions disease prevention as a reason for handwashing	109	67.3	93	59.2	−8.0%	0.149	−19.0%	2.9%
Mentions diarrhea prevention as a reason for handwashing	3	1.9	3	1.9	0.1%	0.957	−3.1%	3.3%
Hygiene advice								
Received health advice from child (prompted)	3	1.9	4	2.6	0.6%	0.711	−2.7%	4.0%
Received health advice from school (prompted)	2	1.2	1	0.6	−0.6%	0.58	−2.6%	1.5%
Received health advice from Accredited Social Health Activist/angawadi (prompted)	5	3.1	6	3.8	0.8%	0.672	−2.9%	4.5%
Was advised to wash hands (unprompted)	13	8.0	14	8.9	0.7%	0.828	−5.9%	7.4%
Was advised to wash hands with soap (unprompted)	10	6.2	12	7.6	1.4%	0.648	−4.6%	7.4%
Exposure to campaign								
Heard about handwashing campaign (prompted)	18	11.4	44	28.6	16.7%	0.001	6.6%	26.9%
Mentions Lifebuoy, Bihar handwashing campaign, or school of 5 campaign (unprompted)	9	5.7	26	17.1	11.1%	0.005	3.4%	18.9%
Heard about mothers meeting (prompted)	3	1.9	18	11.7	9.6%	0.004	3.0%	16.3%
Took part in mothers’ meeting (prompted)	1	0.6	14	9.1	8.3%	0.004	2.6%	14.1%
Took part in mothers’ pledge (prompted)	0	0.0	8	5.2	5.2%	–	–	–
Heard or saw mom’s story (prompted)	2	1.3	14	9.2	7.9%	0.004	2.5%	13.3%

CI = confidence interval.

* Prevalence difference adjusted for village-level clustering (generalized estimating equations/robust standard errors).

When asked after the structured observation about the purposes of the study, 40% of mothers thought the study was about observing food preparation (no difference across arms), whereas 37% thought it was about observing house cleaning (no difference across arms). Only 19 of 322 mothers (6%) thought the purpose was to study handwashing behavior (15 in the control arm and four in the intervention arm, *P* = 0.03).

Of 169 observer/village combinations in the intervention arm, observers guessed the correct allocation of the village (i.e., intervention) in 55.3%. In the control arm, of the 169 observer/village combinations, observers incorrectly guessed in 57.4% that the control village was an intervention village, that is, observers showed a similar tendency in intervention and control villages to regard a village as an intervention village.

## DISCUSSION

The results suggest that the School of 5 campaign, done across the Indian state of Bihar, had little effect on the target behaviors (HWWS after defecation and before each meal, and soap use for bathing) at home among the intended main beneficiaries of the program, school-aged children, and their mothers who also had a child less than 5 years of age. The main intention of the program was to reduce morbidity and mortality in children less than 5 years of age living in households of target schoolchildren, through an assumed behavior and knowledge transfer from schoolchildren to other household members (especially mothers) and possibly a reduced transmission of infections from the schoolchild to younger siblings.^21^ The findings of our trial suggest that little such transfer occurred and that, consequently, a reduction in under five morbidity and mortality was unlikely.

There was an increased awareness of important handwashing occasions in children when asked generally about hygiene and handwashing, without mentioning the campaign. Even so, direct campaign exposure was incomplete in children and especially in mothers, who had notably low knowledge of the campaign and its components. Process evaluation conducted post hoc by a separate team of investigators in four newly recruited intervention schools, and villages revealed that campaign failure may stem from an ineffective delivery of key messaging. Although the live delivery by professional promoters created enthusiasm among students, the largely didactic methods mimicked normal educational approaches in India, focusing on and rewarding repetition and memorization instead of the importance and actual practice of HWWS. This may have made the intended messaging seem like just another topic for the students to remember and may have failed to create an emotional response to the content presented that could drive behavior change toward practicing HWWS.^23–25^ Campaign failure may have also been due to low campaign intensity, especially among mothers, who mostly did not attend events targeted at them. Campaign delivery may have been made difficult by the specific socioeconomic and cultural conditions in Bihar, which is a relatively poor and underdeveloped state of India. This is to some extent supported by the higher effect on handwashing observed in Desari Block, which is more urbanized and closer to the capital, and therefore may be more suitable for public health campaigns than Bibhutipur. The statistical strength of the effect modification by block was however low. Furthermore, the evaluation suggested that children in this setting traditionally may be unlikely to influence behavior at the household level, as at this age, their social position in the household may generally be regarded as weak. The findings demonstrate the difficulty in translating the concept of children as agents of change in the community (which may work on a small scale^18,19,21^) into a large program aiming at reaching nine million children. Further details of the process evaluation will be presented in a separate article. To date, scaling up of hygiene promotion has remained a challenge. A large-scale program evaluated in a CRT in Peru found substantial increases in observed HWWS, but observations were carried out the day following administration of a detailed questionnaire on handwashing behavior which may have caused reactivity in the observed households.^26^ A further CRT embedded in a larger handwashing promotion program in Tanzania where, like in our study, observations that preceded interviews found no evidence for behavior change.^27^

Possible limitations of the study include the method of outcome assessment, the possibility of contamination, incomplete intervention rollout, and imbalance in water access across arms. Direct structured observation of handwashing carries the risk of reactivity, as suggested by the striking contrast in the estimated effects found in the trial in Peru and Tanzania described earlier. Study participants may change their behaviors when they know they are being observed, potentially leading to an overestimate of socially desirable behaviors such as HWWS.^28^ Bias can arise if reactivity is higher in the intervention than in the control arm. We used four methods to minimize the risk of bias in observed HWWS. 1) We did not conduct a baseline survey in the study population, as being surveyed twice with an intervention happening in between may allow study participants to link the study to the intervention. 2) We recruited participants and conducted observations at least 8 weeks after the intervention. 3) We blinded the study team to intervention allocation. 4) Study participants were told a cover story to mask the true purpose of the observation. Although baseline data may have been useful for power calculation and conducting restricted randomization, we believe avoidance of bias is of higher importance in this type of studies. Overall, reported beliefs of treatment allocation among staff and of the purpose of the observations among mothers suggest that bias was minimized to the extent possible in these circumstances.

**Figure 2.**
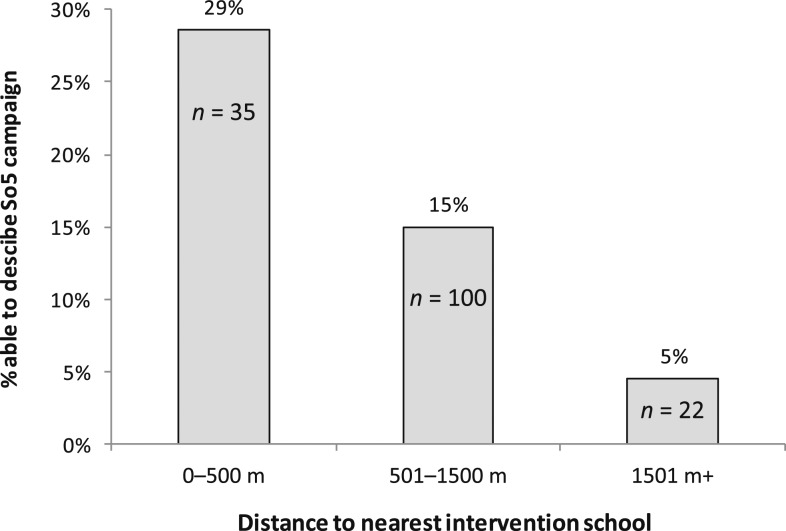
Percentage of children in control schools able to describe So5 campaign, relative to the distance (m) of their school to the nearest intervention school. Note that these children went to 23 different schools (*N* = 4 schools in the shortest, *N* = 15 in the middle, and *N* = 4 schools in the longest distance category).

Although the number of involved schools is too small for robust statistical analysis, Figure 2 suggests that contamination may have occurred across trial arms within a radius of about 1.5 km. Overall, 16% of control children knew of the campaign and could describe aspects of it, possibly via the task for intervention children to tell at least 10 friends or family members about the program. While community intervention studies differ greatly in context and content, our findings suggest that to avoid contamination in similar behavior change trials, intervention and control clusters should be chosen to be no closer than 3 km from each other. Contamination, incomplete intervention rollout, and the slightly worse water access in the intervention arm may have biased any intervention effect toward no effect. We therefore calculated the CACE, adjusted for water access, which suggested that the true effect of the intervention in this population may have been a 7% point rise in HWWS prevalence at target occasions. This effect size was still lower than the intended 15%. Although it cannot be excluded that the School of 5 campaign may have achieved worthwhile educational goals and may encourage children to practice hygiene behaviors later in life, there was no relevant immediate effect on HWWS with the potential to reduce transmission of infections in the home, especially to vulnerable children less than the age of 5 years, the intended main program beneficiaries.

## Supplementary Material

Supplemental table
